# In defence of the three-domains of life paradigm

**DOI:** 10.1186/s12862-017-1059-z

**Published:** 2017-09-19

**Authors:** P.T.S. van der Gulik, W.D. Hoff, D. Speijer

**Affiliations:** 1 Centrum Wiskunde & Informatica, P.O. Box 94079, 1090 GB Amsterdam, Amsterdam, The Netherlands; 2 Department of Microbiology and Molecular Genetics and Department of Chemistry, Oklahoma State University, Stillwater, OK 74078 USA; 3 Department of Medical Biochemistry, Academic Medical Center, University of Amsterdam, Meibergdreef 15, 1105 AZ Amsterdam, Amsterdam, The Netherlands

**Keywords:** Eucarya, LECA, Phylogenetics, Eukaryogenesis, Three-domain model

## Abstract

**Background:**

Recently, important discoveries regarding the archaeon that functioned as the “host” in the merger with a bacterium that led to the eukaryotes, its “complex” nature, and its phylogenetic relationship to eukaryotes, have been reported. Based on these new insights proposals have been put forward to get rid of the three-domain Model of life, and replace it with a two-domain model.

**Results:**

We present arguments (both regarding timing, complexity, and chemical nature of specific evolutionary processes, as well as regarding genetic structure) to resist such proposals. The three-domain Model represents an accurate description of the differences at the most fundamental level of living organisms, as the eukaryotic lineage that arose from this unique merging event is distinct from both Archaea and Bacteria in a myriad of crucial ways.

**Conclusions:**

We maintain that “a natural system of organisms”, as proposed when the three-domain Model of life was introduced, should *not* be revised when considering the recent discoveries, however exciting they may be.

## Background

The discovery that methanogenic microbes differ fundamentally from Bacteria such as *Escherichia coli* or *Bacillus subtilis* constitutes one of the most important biological discoveries of the twentieth century. These revolutionary insights regarding the nature of prokaryotes resulted from the analysis of ribosomal (r) RNA sequences by Carl Woese, unambiguously demonstrating that they consist of two entirely distinct cells: Bacteria and Archaea [1]. rRNA analysis allowed retention of the clear dichotomy based on ultrastructure found in electron microscopic studies of cells: prokaryotes with simple structures on the one hand and nucleated cells with complex intracellular compartments and membrane systems, on the other. The dichotomy based on ultrastructural analysis remained essentially valid after the discovery of the Archaea. Partly because the scientific community was slow in recognizing the momentous discovery, Woese decided to present his discovery in a more forceful way, by formally introducing *regio* (English: domain) as a higher taxonomic level than *regnum*: introducing the three domains of cellular life, Archaea, Bacteria and Eucarya. This paradigm was proposed by Woese, Kandler and Wheelis in PNAS in 1990 [2]. Acceptance (slowly) followed, and today the three-domain model is taught in virtually all biological textbooks, and routinely treated as a given in biological research and publications.

Recently, evidence has been accumulating (see e.g. [3, 4] and references therein) that the Eucarya (members of which shall be referred to as “eukaryotes” in the remainder of the text) may “just” be the result of a merger of an archaeon and a bacterium. Now, the discoveries of new phyla of Archaea more closely related to the “host” in this scenario than found ever before, are indeed of major importance and the composite genomes contain some highly interesting extra “eukaryote-specific” genes. Thus the archaeal host already seems to have had some important components for later eukaryotic cell complexity. Equally important, however, is the observation that these cells are in no way “pre-eukaryotes”, missing many other key pieces that will come from the bacterial endosymbiont and others that can only be classified as “eukaryotic inventions” so far; see e.g. [5, 6].

The authors of the studies regarding the relevant host-related Archaea started an important discussion regarding the most appropriate view of the taxonomy of cellular life. Their genome findings are quoted by some as a reason to abandon the three-domain model, and replace it by a two-domain model. First, in 2013, Embley and co-workers published an article entitled: “An Archaeal Origin of Eukaryotes supports only two primary domains of life” [7]. Next, in 2015, Raymann, Brochier-Armanet and Gribaldo already used the alternative quite matter-of-factly in the title of their publication “The two-domain tree of life is linked to a new root for the Archaea” [8]. A quote from this article: “Collectively, the results from the A/E and A/B analyses support a two-domain topology for the tree of life, with eukaryotes as a sister lineage to the whole TACK superphylum” [8]; N.B. “TACK superphylum” refers to the archaeal superphylum comprising the phyla Thaumarchaeota, Aigarchaeota, Crenarchaeota and Korarchaeota. This seems to encourage taking away the domain-level from the Eucarya. Then, also in 2015, Koonin stated: “…the results of increasingly robust phylogenomic analyses appear to be best compatible with a model of two primary domains of cellular life, Archaea and Bacteria, as opposed to the standard three-domain model” [9]. Last, but not least, in the latest description of the new phyla of Archaea, Zaremba-Niedzwiedzka and co-workers also describe their findings as “...reinforcing the validity of the two-domain topology of the tree of life…..” [4]. We understand the “host-endosymbiont view”, and the concept of the eukaryote as a “modified host” still belonging to the Archaea, but consider it inappropriate.

It might be useful to sketch the frame of reference for these kinds of pronouncements. Since Darwin started drawing phylogenetic trees, biological taxonomy has been dominated by phylogeny, with present-day organisms sharing common ancestors. In this classical view the fusion of distinct phylogenetic branches does not occur. Horizontal gene transfer (HGT), and especially the process of endosymbiosis thus poses major problems, requiring important decisions on how to classify organisms in which fusing branches play a (major) role. Initially, this issue was largely ignored with regard to eukaryotic origins because of the following view: that they started out as pre-endosymbiotic (presumably anaerobic), phagocytosing, cells. The picture of such an “anaerobic amoeba” feeding on Bacteria, sits well with the tendency towards linear phylogenetic branching. However, such a picture is deeply flawed (see the next sections). The “host-endosymbiont view” suggests that eukaryotic cells are merely modified forms of the cell that acquired the bacterial component, which blinds us to the bacterial contributions to the Eucarya. We will come back to this, but first focus on why basal taxonomy is important.

## Why defend the three-domains of life paradigm?

Here, we concisely try to defend the three-domain model, not because we do not agree with the concept that the eukaryotes came about as the result of a merger of an archaeon and a bacterium (we emphatically *do* agree), but because the eukaryotic cell is *not* an archaeon with a bacterium inside, and because we are convinced that at the most fundamental level there are three cell types, archaeal, bacterial and eukaryotic, and that the primary biological distinction should reflect this reality. At this point we also want to stress the fact that these taxonomic considerations are not “just about semantics”. Taxonomy (especially at this most fundamental level) should not only reflect our scientific findings as accurately as possible, but will shape their interpretation in turn. To quote Woese: "A biological classification is...an overarching evolutionary theory that guides our thinking and experimentation, and it must be structured...to reflect evolutionary reality" [10]. In the article quoted he reacted to the objections Ernst Mayr made against raising the Archaea to the domain level. Mayr, using morphological (“phenetic”) criteria found the differences between eukaryotes and “prokaryotes” (Archaea *and* Bacteria) more important, and thus argued for a two-domain (“Empire”) model [11]. One might think that the present discussion just recapitulates this argument between Woese and Mayr in the mid 1990s, however there are major differences. First of all, the latest two-domain model is along the Archaea Bacteria divide with the Eucarya forming part of the Archaea. Secondly, there is no discussion about the primacy of the phylogenetic (“cladistic”) approach, but we contend that the complete merging of cells and genomes at the basis of the eukaryotes places it outside the normal phylogenetic framework.

## A naive host-endosymbiont framework distorts our idea of eukaryogenesis

If we are to succeed in our aim of distinguishing three fundamental cell types, we first have to get rid of a most harmful preconception. The picture which is generally present (though as a tacit assumption) when discussing eukaryogenesis, is that of a large, “pre-eukaryote” or “pre-eukaryotic archaeon” which takes up a small, alphaproteobacterial cell, which is subsequently reduced to an organelle. With this picture in mind, it is only natural to state: “Collectively, the results from the A/E and A/B analyses support a two-domain topology for the tree of life, with eukaryotes as a sister lineage to the whole TACK superphylum”. An important first issue ignored here, is the *huge* amount of eukaryotic genes with a bacterial origin. As Koonin observes: “Notably, altogether, the number of eukaryotic protein-coding genes of bacterial origin exceeds the number of ‘archaeal’ proteins about threefold” [9].

The distorting nature of this picture, preventing understanding of eukaryogenesis, is perfectly illustrated by ribosome structure. The *larger* kind of ribosomes present in the cytosol should not be seen as “*the* eukaryotic ribosomes”, contrasted with the “bacterial” ribosomes in the mitochondrion. *Both* the ribosomes in the cytosol and the ones in the mitochondrion are eukaryotic. The cytosolic ribosome is different from prokaryotic ribosomes in many respects. Eukaryotic cytosolic ribosomes are much larger and more complex, incorporating more rRNA (the so-called expansion segments) and extra ribosomal proteins or protein extensions. This goes along with some fundamentally different activities and regulations (see e.g. [12]). However, the mitoribosome is just as different from the bacterial one, although in other respects. They instead are characterized by inverting protein-to-RNA ratios (reducing rRNA while gaining extra proteins). These changes reflect both pressures of overall mitochondrial DNA reduction and the requirements of mitochondrial protein synthesis: production and membrane delivery of hydrophobic proteins. See e.g. the characterization of the human mitoribosome [13] and references therein. Thus: *the eukaryotic cell is not the ancestral archaeal host with minor modifications and the mitochondrion is not a reduced bacterium.* Having freed us of the misleading image of a sister of TACK swallowing up a bacterium, even though the term “host” makes this difficult to do, we are more inclined to appreciate the remarkable fact that at a rather late stage (~ 2 billion years ago, compared with the ~3.8 billion years of prokaryotes) of life’s evolution *a truly new type of cell emerged* (see next paragraph). Of note, the fact that many of the “genes of bacterial origin” Koonin referred to, were already present in the archaeon before the merger (see e.g. [14]) at the basis of the eukaryotic tree is not relevant to our argument.

## Symbiogenesis dictates a three-domain model of life

In 1998 another important paper regarding evolution at a very fundamental level appeared: Martin and Muller proposed the "The hydrogen hypothesis for the first eukaryote" [15]. This hypothesis launched symbiogenetic theory: the idea that defining eukaryotic characteristics came about *as a result of mutual adaptations of two prokaryotes.* Whether one agrees with the specifics of the proposal is less important than this conceptual framework. More and more new findings demonstrate that this general scheme is basically correct; (see e.g. [16] and references therein). We will highlight just three examples of the eukaryotic features which only make sense in the light of symbiogenetic theory. First of all, the mutual adaptation has led to one of the quintessential eukaryotic characteristics, the ability to generate large amounts of ATP to “pay” for the energy consuming eukaryotic inventions [17]. Secondly, one of these inventions, the complete eukaryotic endomembrane system, including the nuclear membrane, probably arose out of bacterial outer membrane vesicles released by the mitochondrial ancestor into the cytosol [5], a model which also explains the replacement of the archaeal (host) membrane lipids by bacterial ones (see later). Lastly, peroxisomes are best understood as organelles which evolved as a system to alleviate internal ROS formation associated with (pre)mitochondrial fatty acid oxidation [18, 19]. Apart from these attributes, e.g. metabolic restructuring, reactive oxygen species (ROS) signalling, meiotic sex and autophagy all are likely to have come about as a result of the merger. The presence of sufficient oxygen only after the great oxygenation event (GOE), and its requirement for eukaryotic evolution, both because of highly efficient ATP generation and as a “creative force” in the form of ROS, have already implicitly been indicated here; see [20] and references therein. We would like to stress the fact that both participants contributed equally to the final eukaryotic cell. This initiated a *highly synergistic process* in which the mutually adapting partners evolved into much more than the sum of their parts [16]. Another way of looking at this: eukaryote evolution is starkly characterized by “emerging properties”, which make up the greatly increased cellular complexity. Describing eukaryotes as part of a “sistergroup of the TACK superphylum” thus flies in the face of symbiogenetic theory. The details of the symbiogenetic process (which for a large part are still a matter of speculation) we do not consider relevant for what is at issue here; however, some possible models can be found in [5, 6, 15, 16].

## Genome biology: Further support for the three-domain model from three types of tRNA sets

We again introduce a “milestone paper”: in 2012 Novoa et al. described a major role for tRNA modifications in codon usage in the different domains of life [21]. In this study they showed the eukaryotic “absence” of a correlation between tRNA gene content (a proxy for tRNA concentration) and codon usage to be only superficial. This incorrect impression was due to ignoring wobble rules; in earlier work, researchers were looking for correlation using regular Watson-Crick pairing only. Furthermore, Novoa et al. *showed that each of the primary domains has its own typical codon usage as a result of domain-specific anticodon modification patterns*. What are the domain-specific characteristics? In short, in eukaryotes inosine (the product of a tRNA-dependent adenosine deaminase) is used in many tRNA anticodon first-positions, in the Archaea inosine is not used at all, and in Bacteria inosine is only used in the anticodon first position in one codon box (CGN). *This use determines domain-characteristic codon usage patterns.*

Of note, eukaryotes generally have (at least) a double tRNA set as the mitochondrial genome (almost always) encodes a second set of tRNAs. The domain-characteristic codon usage pattern of eukaryotes referred to above is linked to the cytosolic tRNAs. Considering domain-specific codon usage and tRNA gene content characteristics, a further factor is the specific bacterial modification of U into xo^5^U by tRNA-dependent uridine methyltransferases, which also leaves its “fingerprint” on *bacterial* codon usage. How do these fingerprints come about? The evolution of tRNA repertoires and codon use can be understood in light of the fact that modifications of the wobble position increase the decoding capacity of tRNAs, and thus, crucially, translation efficiency. Modifiable tRNAs are then positively selected (and tRNA gene content distribution/codon usage with it). As the modification enzymes differ between the three domains we find a characteristic codon usage bias for each [21]. We are surprised that this basic, crucial difference between the three domains does not get wider recognition, especially as it shows that at a relatively late stage in evolution - i.e. at the birth of the eukaryotes - a *signature* eukaryotic codon usage pattern could still evolve (due to the substrate repertoire expansion of a tRNA-dependent adenosine deaminase). We quote: “One functional theme that has emerged is that the addition of a partner protein may allow for an expansion of the substrate repertoire for a given enzyme, as for the eukaryotic Tad2-Tad3 enzyme, which modifies an additional 6 tRNA species (in *S. cerevisiae*) in contrast to its homodimeric bacterial TadA counterpart, which appears to only modify tRNA^Arg(ACG)^” [22].

Thus, not only in cell structure, membrane composition and general level of complexity, do we find three kinds of cell. Also in tRNA set (as defined above) and codon usage (so, in the basic genetic structure) the living world is divided three ways. This basic three way partition should be reflected in the primary taxonomic division, and after starting with that, phylogeny can step in to provide the details per domain. Of note, chloroplasts do not complicate this picture. They come from a cyanobacterium entering at a later stage (after the arrival of the Last Eukaryotic Common Ancestor (LECA)). The endosymbiosis leading to green algae is a major event, but not comparable in impact with that of eukaryogenesis. Another way of describing the difference: in the event leading up to LECA, an archaeon combined with an alphaproteobacterial cell to give rise to a eukaryote (which is *not* an archaeal cell), in the event leading up to green algae, a eukaryote took in a cyanobacterial cell, while remaining eukaryotic.

## Further considerations

In the context of ideas regarding the domains of life, a few other observations have to be discussed. Though never popular, recent discoveries of giant viruses, such as the mimivirus [23], have given rise to the idea of a fourth domain of life. There has even been speculation that they played a crucial part in the origin of eukaryotes (e.g. contributing the structures at the origin of the nucleus [24]). These ideas were criticised upon further analysis: ".... there is no solid evidence for the existence of a viral domain of life or for a significant implication of viruses in the origin of the cellular domains" [25]. More recently, discovery and analysis of giant Klosneuviruses in metagenomic data showed them to be derived from smaller viruses vastly supplied by host genes [26], reinforcing the conclusion that in these cases, cellular homologues in viruses result from HGT *to* their infecting viruses rather than the opposite [25].

We have used the term “prokaryote”, referring to both Archaea and Bacteria, rather freely. Norman Pace objects to the use of the term, which he (correctly) interprets to be a negatively defined concept (“not being a eukaryote”), developed prior to the insight regarding the chasm between Archaea and Bacteria [27]. It might be even the case that Archaea and Bacteria do not share a common *cellular* ancestor (a scenario which we will not discuss here; see e.g. [28]). We use “prokaryote” as a functional description, not as a phylogenetic one. However, we also think it a useful term, as Archaea and Bacteria are both more ancient and simple cellular entities, clearly different from the cell type resulting from the process of eukaryogenesis, which can be considered as a revolutionary union of an archaeon and a bacterium.

## Conclusion: The three-domain model of life best reflects biological reality and should thus be retained

Because of the considerations brought forward here, we propose to *retain* the three-domain model, with the understanding that the primary taxonomic division among cellular organisms ought to reflect that 3 basic cell types exist. First, an archaeal cell type, with isoprenoid units attached to glycerol-1-phosphate by ether linkages forming their membranes and a codon usage not shaped by inosine. Then, another prokaryote, the bacterial cell type with ester-linked lipids (fatty acids linked to glycerol-3-phosphate by ester linkages) and a codon usage mainly moulded by a bacterial tRNA dependent uridine methyltransferase. Finally, the complex cell type resulting from eukaryogenesis, the type representing the “Eucarya” of Woese, Kandler and Wheelis. Here codon usage reflects the massive use of inosine in anticodons, membranes are of the bacterial type, two types of ribosomes -*both* different from prokaryotic ones- are present, and a completely reconfigured metabolism resulting from the interactions between both prokaryotes involved, can be found. Later on, some eukaryotes even acquired further ribosomes (e.g. in chloroplasts). In conclusion, our proposal does *not* challenge the primacy of the phylogenetic approach, and allows the first taxonomic division to be a *functional* division, reflecting biological reality. Three phylogenetic trees can now be build, separately, for each of the domains. We have to start with a tiny Darwinian forest before we can tend to its massive trees.
